# Bacterial Disinfection by CuFe_2_O_4_ Nanoparticles Enhanced by NH_2_OH: A Mechanistic Study

**DOI:** 10.3390/nano10010018

**Published:** 2019-12-19

**Authors:** Yu Gu, Furen Xiao, Liumin Luo, Xiaoyu Zhou, Xiaodong Zhou, Jin Li, Zhi Li

**Affiliations:** 1 School of Mechanical and Eletrical Engineering, Zhoukou Normal University, Zhoukou 466000, China; bird_llm@163.com (L.L.); vzhouxy@163.com (X.Z.); zhouxd516@163.com (X.Z.); lijin309@126.com (J.L.); 2 College of Materials Science and Engineering and State Key Laboratory of Metastable Materials Science and Technology, Yanshan University, Qinhuangdao 066004, China; frxiao@ysu.edu.cn; 3 California State University San Bernardino, 5500 University Pkwy, San Bernardino, CA 92407, USA; liz312@coyote.csusb.edu

**Keywords:** copper ferrite, hydroxylamine, Cu(I), reactive oxygen species, water pathogen

## Abstract

Many disinfection technologies have emerged recently in water treatment industry, which are designed to inactivate water pathogens with extraordinary efficiency and minimum side effects and costs. Current disinfection processes, including chlorination, ozonation, UV irradiation, and so on, have their inherent drawbacks, and have been proven ineffective under certain scenarios. Bacterial inactivation by noble metals has been traditionally used, and copper is an ideal candidate as a bactericidal agent owing to its high abundance and low cost. Building on previous findings, we explored the bactericidal efficiency of Cu(I) and attempted to develop it into a novel water disinfection platform. Nanosized copper ferrite was synthesized, and it was reduced by hydroxylamine to form surface bound Cu(I) species. Our results showed that the generated Cu(I) on copper ferrite surface could inactivate *E. coli* at a much higher efficiency than Cu(II) species. Elevated reactive oxygen species’ content inside the cell primarily accounted for the strong bactericidal role of Cu(I), which may eventually lead to enhanced oxidative stress towards cell membrane, DNA, and functional proteins. The developed platform in this study is promising to be integrated into current water treatment industry.

## 1. Introduction

Water pathogens are a great concern that threaten the safety of public drinking water. It has been reported that outbreaks of mass diseases in the cities are most likely linked to the failed disinfection facilities [1,2,3]. So far, water disinfection has been a widely researched topic, but nonetheless, available water disinfection techniques are limited. Most of the currently used water disinfection methods are chlorination, ozonation, and UV irradiation, and so on, all of which have significant drawbacks [4,5,6,7,8]. For example, chlorination byproducts after reaction with organic compounds in water are reported to be carcinogenic and are not generally avoided around the world [9,10]. Ozonation is a rather clean and powerful method, however, the electrochemical production of ozone relies on special anode materials and high applied voltage to surpass the overpotential of ozone evolution. Moreover, toxic nitric oxide species might also be generated during the electrochemical production of ozone [11,12]. Compared with the above two methods, UV irradiation is relatively simple and less vigorous, because lights in the UV range could penetrate the cell membrane to impair DNA and cause gene breakdown. While demonstrated in clear water bodies such as potable water, UV irradiation is ineffective in dark water bodies as the lights’ travel is blocked [8]. In addition, it has to be noted that water pathogens are observed to develop resistance to those traditional disinfection technologies, including chlorination/chloramination [13,14,15], ozonation [16,17,18], and UV irradiation [19,20,21]. Therefore, the development of more potent and environmentally-friendly techniques is necessary.

Recently, multifarious nanomaterials have been developed for bacterial inactivation purposes. For instance, a recent study reported that g-C_3_N_4_ nanolayers under visible light could kill *Escherichia coli* with high efficiency by generating reactive oxygen species [22,23]. However, the mass production of high quality g-C_3_N_4_ nanolayers has not been achieved so far, impeding the application of this material in the water disinfection industry. Graphene-based nanoparticles as a disinfectant have also attracted numerous attention [24,25], but face the same issue of upscaled production. Another widely used antibacterial reagent belongs to the silver-based material family [26,27,28]. Silver inactivates bacteria mainly through binding to the thiol groups of functional proteins and destroying the protein native structure [27]. The issue that prohibits silver from large-scale application into water disinfection industry is its high cost. 

Copper has been used as an antibacterial material of low cost and easy manufacturing for hundreds of years [29,30,31]. It has been revealed that copper inactivates bacteria through a mechanism similar to silver’s bactericidal role, that is, deactivating functional proteins by chelating thiol groups [27]. Further studies showed that, among all copper valency (Cu(0), Cu(I), and Cu(II)), Cu(I) owns the highest antibacterial activity, mainly because Cu(I) has strong binding affinity, as well as its reduction capability towards functional proteins [7]. These two synergistic functions of Cu(I) lead to its bacteria inactivation performance with hundreds of times higher efficiency than Cu(II) and Cu(0). It has been previously reported that Cu(II) could be reduced by hydroxylamine (NH_2_OH) to produce Cu(I) with a high efficiency [32,33], but Cu(II) ion should be prohibited from drinking water because of its strong toxicity to human beings [34,35]. Specifically, the U.S. Environmental Protection Agency (EPA)-permitted copper ion concentration in drinking water is 1.3 ppm [36]. Herein, we demonstrate that heterogeneous copper ferrite (CuFe_2_O_4_) nanoparticles with minimum leached toxic copper ions, after reduction by hydroxylamine, show significantly higher antibacterial activity than that without hydroxylamine addition. In this study, we chose *E. coli* as the target species, mainly because it has wide infectivity in various water bodies [37,38]. The bactericidal mechanism by CuFe_2_O_4_/NH_2_OH was also revealed in this study with several molecular probes. Overall, this controllable manner of chemical addition and powerful bactericidal performance could attract the attention of the water disinfection industry.

## 2. Materials and Methods 

### 2.1. Materials

CuSO_4_ (Alfa Aesar, Ward Hill, MA, USA), Fe_2_(SO_4_)_3_•5H_2_O (ACROS Organics, Morris Plains, NJ, USA) and dodecyltrimethylammonium bromide (Sigma Aldrich, Natick, MA, USA) were used to synthesize CuFe_2_O_4_ nanoparticles. Hydroxylamine (NH_2_OH) was purchased from Sigma Aldrich (Natick, MA, USA). LB medium and agar from BD Difco (Pittsburgh, PA, US) were used to culture *E. coli* cells. MOPS buffer (ACROS Organics, Morris Plains, NJ, USA) was used to maintain the physiological integrity of cell membrane. HPF probe (3’-p-(hydroxyphenyl) fluorescein, Thermo Fisher Scientific, Bedford, MA, USA) and EDTA (Millipore Sigma, Burlington, MA, USA) were used to scavenge ROS (reactive oxygen species) generation. Milli-Q water was used throughout the study, and all nutrients were autoclaved before being used to culture *E. coli* cells.

### 2.2. Synthesis and Characterization of Copper Ferrite

We followed a reported procedure to synthesize copper ferrite nanoparticles [39]. Briefly, 0.1 M dodecyltrimethylammonium bromide was used as capping agent. Then, 1.6 g CuSO_4_ and 4.9 g Fe_2_(SO_4_)_3_•5H_2_O was added into the solution to achieve a Fe/Cu molar ratio of 2:1. The solution was stirred by a magnetic bar for 15 min to totally dissolve copper and iron salts. Then, solution pH was adjusted to pH 12.5 with 5 M NaOH, and the solution was then stirred for 45 min to allow sufficient precipitation. Subsequently, the solution was transferred into an autoclave vessel, and kept at 120 °C for 1 h. After hydrothermal treatment, the obtained powders were then extensively washed with hexane. Eventually, the powders were sintered in a 100 °C oven overnight. The produced CuFe_2_O_4_ nanoparticle samples were collected for future use.

Copper and iron ions concentration in solution were quantified with ICP (inductively coupled plasma). Then, 2% HNO_3_ solution was used to dissolve particles. Copper and iron were analyzed with emission wavelengths of 324.754 nm and 259.940 nm, respectively.

The synthesized CuFe_2_O_4_ nanoparticles were then characterized by transmission electron microscopy (TEM, JEOL, Beijing, China). The crystal structure was analyzed by X-ray diffractometry using a Thermo Scientific ARL EQUINOX 1000 diffractometer, and X-ray photoelectron spectroscopy studies were performed utilizing a Thermo Scientific™ K-Alpha™ spectrometer to evaluate the electronic properties of elements on the surface of synthesized CuFe_2_O_4_ nanoparticles.

### 2.3. *E. Coli* Inactivation Assay

Exponential phase *E. coli* cells were used for the bacterial inactivation assays. A single *E. coli* K12 colony picked up from an LB-agar petri dish was cultured overnight in 5 mL LB medium at 37 °C. Then, 50 µL saturated *E. coli* cell solution was added into 5 mL fresh LB medium. The bacterial solution was then shaken at a speed of 250 rpm at 37 °C for around 2 h, until OD600 reached ~0.7. Exponential phase *E. coli* cells were then collected by centrifugation at 5 g for 1 min, and washed extensively with 10 mM MOPS buffer (pH 7) to remove residual nutrients. Subsequently, cells were transferred in 5 mL of 10 mM MOPS buffer and stored at 4 °C. Bacterial solutions were used within the same day.

In a typical antibacterial assay, MOPS buffer was replaced by a solution containing 0.2 g/L CuFe_2_O_4_ and 2 mM NH_2_OH with *E. coli* cells. The solutions were constantly shaken at 37 °C at a speed of 250 rpm. At each hour, 200 µL solution was withdrawn from the tube for analysis, and the bacteria survival rate was determined by a 10-fold serial dilution method in 96-well plates [7]. A volume of 10 µL from the six dilutions of each sample was dropped onto an LB-agar plate, and incubated at 37 °C overnight. To calculate the total survived cell number, CFUs (colony forming units) were counted.

In order to investigate whether the bactericidal role of CuFe_2_O_4_/NH_2_OH was from the reaction between leached Cu^2+^ and NH_2_OH, we performed a simulation experiment. It was determined that the leached Cu^2+^ concentration in solution was below 20 ppb. Therefore, 20 ppb Cu^2+^ was mixed with 2 mM NH_2_OH, and bactericidal efficiency was then determined with the abovementioned assay.

### 2.4. ROS Quantification Assay

The ROS content in *E. coli* cells was quantified by a fluorescent HPF probe. In detail, bacterial cultures were incubated with 10 µM HPF at 37 °C. At 1, 2, and 3 h, and 200 µL was transferred into a 96-well plate for analysis. The fluorescence analysis was performed with excitation/emission maxima at 490/515 nm, respectively. The following positive and negative groups were researched [40]: (1). 0.2 g/L CuFe_2_O_4_, 2 mM NH_2_OH, with *E. coli* cells; (2). 0.2 g/L CuFe_2_O_4_, with *E. coli* cells; (3). 2 mM NH_2_OH, with *E. coli* cells; (4). 0.2 g/L CuFe_2_O_4_, 2 mM NH_2_OH, without *E. coli* cells; and (5) *E. coli* cells. The (2)–(5) negative controls were used, to verify that the generated ROS signal in *E. coli* cells after CuFe_2_O_4_/NH_2_OH reaction was indeed from the oxidative stress within bacterial cells. The results indicated that the (2)–(5) negative controls produced negligible fluorescence response (data not shown), suggesting that the used CuFe_2_O_4_ or NH_2_OH chemical or *E. coli* cells have no influence on HPF fluorescence. Therefore, the HPF used in this method is valid for measuring ROS content in our study.

### 2.5. ROS Scavenging Assay

We conducted ROS scavenging assays, in order to verify that ROS played a vital role in inactivating *E. coli* cells. In detail, 2 mM EDTA or 10–100 mM DMSO was added into a bacterial solution containing 0.2 g/L CuFe_2_O_4_ nanoparticles and 2 mM NH_2_OH. Reaction solutions were maintained at 37 °C, and shaken at 250 rpm. The bacterial survival rate was determined by the abovementioned serial dilution method.

### 2.6. Recycling of Copper Ferrite

The reusability of heterogeneous CuFe_2_O_4_ nanoparticles was examined. After each round of aqueous reaction, CuFe_2_O_4_ nanoparticles were collected by centrifugation at 10 g for 5 min. The pellets were then transferred into an oven at 80 °C to heat for 2 h. Afterwards, the pellets were used for the subsequent round of bacterial inactivation assay. 

### 2.7. Statistical Analysis

Bactericidal assays were performed with three independent replicates (*n* = 3), and statistical analysis was performed with *t*-test. Asterisks of *p*-values indicate the level of significance compared with *E. coli* control cells in MOPS buffer, that is, ** *p* < 0.01 and *** *p* < 0.001.

## 3. Results and Discussion

### 3.1. Characterization of Synthesized Copper Ferrite

**Figure 1:**
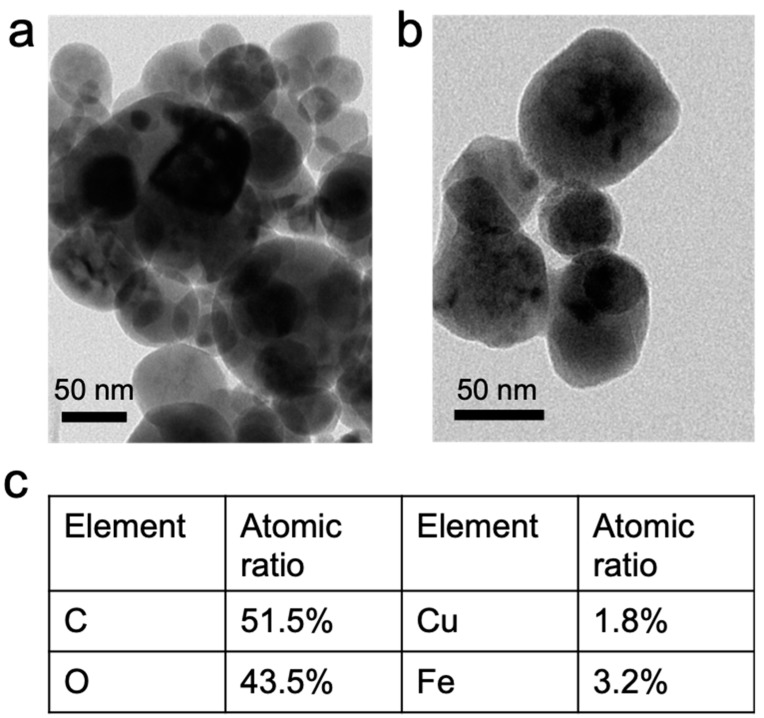
(**a**,**b**) Morphology of synthesized CuFe_2_O_4_ nanoparticles by transmission electron microscopy (TEM), and (**c**) elemental composition of CuFe_2_O_4_ nanoparticles.

**Figure 2:**
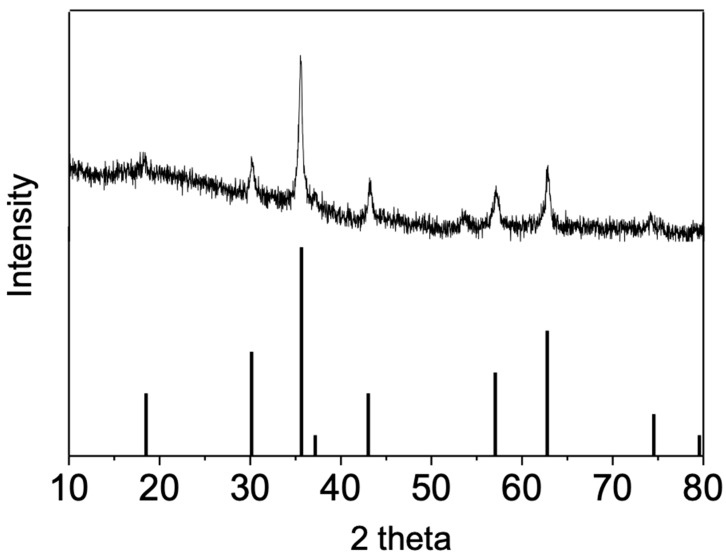
X-ray diffraction (XRD) characterization of synthesized CuFe_2_O_4_ nanoparticles.

The copper ferrite (CuFe_2_O_4_) used was synthesized via a hydrothermal method [39]. After synthesis, the powders were extensively washed to remove residual copper and iron salts from CuFe_2_O_4_ nanoparticles. The residual copper and iron ion concentrations in solution was below 30 ppb measured by ICP, which was primarily because of leaching. The synthesized CuFe_2_O_4_ nanoparticles were then subjected to analysis to confirm their identity. As shown in Figure 1a,b, the CuFe_2_O_4_ morphology was examined by TEM. The CuFe_2_O_4_ nanoparticle sizes were in the range of 20–80 nm, showing relatively homogeneous distribution. The pseudospherical shape of the nanoparticles was owed to the isotropic growth of the crystal from a core. Besides, it is also noted that these nanoparticles tended to aggregate, because of the iron magnetic interactions between particles. It was further determined that the atomic ratio between Cu and Fe is 1.8:1 (Figure 1c), close to theoretical 2:1 value. The detected carbon element was attributed to the used capping reagent and hexane cleaning agent. The crystal structure of CuFe_2_O_4_ was then probed by XRD (X-ray diffraction). It was shown in Figure 2 that the diffraction pattern of obtained sample matched well with the standard, indicating that the main phase of the powder was cuprospinel. In particular, the main peak at 2theta = 35.64 degree dominated both the standard and the obtained sample.

### 3.2. Enhanced Bactericidal Performance of Copper Ferrite by Hydroxylamine Addition

**Figure 3:**
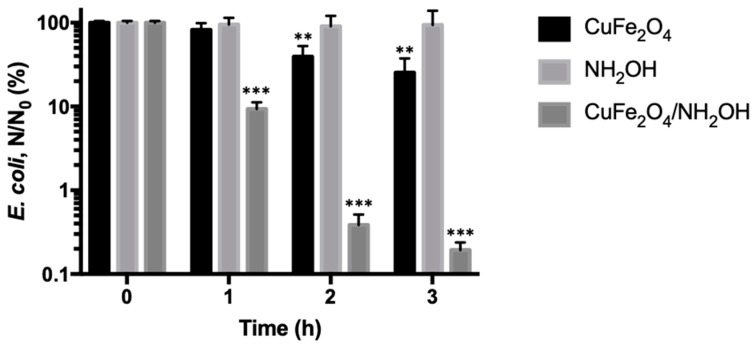
Inactivation of *E. coli* cells by CuFe_2_O_4_ nanoparticles, NH_2_OH, and CuFe_2_O_4_/NH_2_OH. During reactions, 0.2 g/L CuFe_2_O_4_, 2 mM NH_2_OH, and 10^8^ CFU/mL *E. coli* cells in 10 mM MOPS buffer at pH 7 were used. ** *p* < 0.01, *** *p* < 0.001.

We next investigated the bactericidal potential of the synthesized CuFe_2_O_4_ nanoparticles. The exponential phase *E. coli* cells were incubated with 0.2 g/L CuFe_2_O_4_ nanoparticles at 37 °C under shaking conditions, and bacterial viability was measured every hour. It was shown that CuFe_2_O_4_ nanoparticles only induce slight bacterial death during the 3 h incubation, indicating that CuFe_2_O_4_ nanoparticles are a weak antibacterial agent, primarily because of limited surface exposed Cu(II) species (Figure 3). 

It is interesting to observe that, after addition of hydroxylamine (NH_2_OH), the antibacterial potency of CuFe_2_O_4_ nanoparticles increased remarkably. For instance, the *E. coli* cell inactivation rate increased from 0.60-log by CuFe_2_O_4_ nanoparticles to 2.71-log by coupled CuFe_2_O_4_/NH_2_OH reaction after incubation for 3 h. In addition, it was observed that NH_2_OH alone did not show detectable toxicity to *E. coli* cells. Specifically, after incubating exponential phase *E. coli* cells with 2 mM NH_2_OH for 3 h, the cell inactivation rate was 0.03-log. The above results demonstrated that the bactericidal capacity by CuFe_2_O_4_/NH_2_OH reaction was in fact from a new generated species rather than either CuFe_2_O_4_ or NH_2_OH alone. Besides, the bacterial inactivation action by CuFe_2_O_4_/NH_2_OH reaction exhibited a time-dependent pattern, and the *E. coli* cells’ inactivation rate at 1, 2, and 3 h was 1.03-, 2.41-, and 2.71-log, respectively (Figure 3). The progressive bacterial death thus indicated a persistent antibacterial mode by CuFe_2_O_4_/NH_2_OH reaction. It is worth noting that the bacterial amount in drinking water bodies is around 10^4^ CFU per mL, and 99% removal efficiency is desired in most cases [41,42,43]. As the CuFe_2_O_4_ nanoparticles could inactivate 2.71-log of 10^8^ CFU/mL *E. coli* cells after addition of NH_2_OH, the disinfection process developed in this study holds great potential in the streamlined water treatment industry.

**Figure 4:**
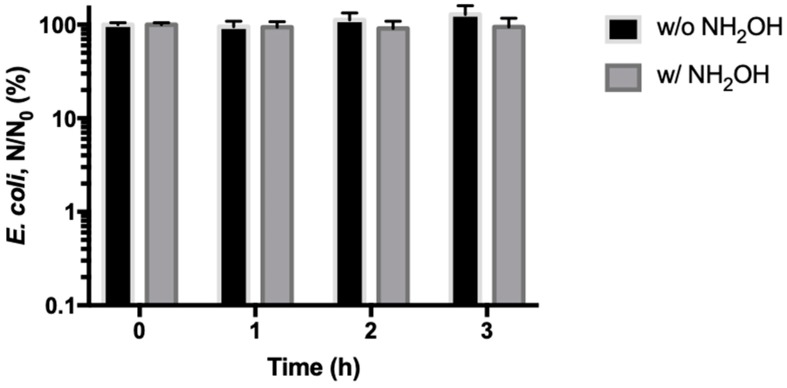
Inactivation of *E. coli* cells by homogeneous Cu(II)/NH_2_OH reaction with or without NH_2_OH. During reactions, 20 ppb Cu(II) ions, 2 mM NH_2_OH, and 10^8^ CFU/mL *E. coli* cells in 10 mM MOPS buffer at pH 7 were used.

It should be noted that the observed strong antibacterial capability of CuFe_2_O_4_/NH_2_OH reaction could be attributed to leached copper ions into the solution. For this purpose, we used ICP to analyze dissolved copper ions in the solution. It was revealed that the detected copper ion concentration was below 20 ppb. To test if such an amount of copper ions could play a role in inactivating *E. coli* cells, we spiked 20 ppb Cu(II) ion into the bacterial solutions with or without 2 mM NH_2_OH. The results showed that the homogeneous Cu(II)/NH_2_OH reaction had no effect on bacterial inactivation (Figure 4).

**Figure 5:**
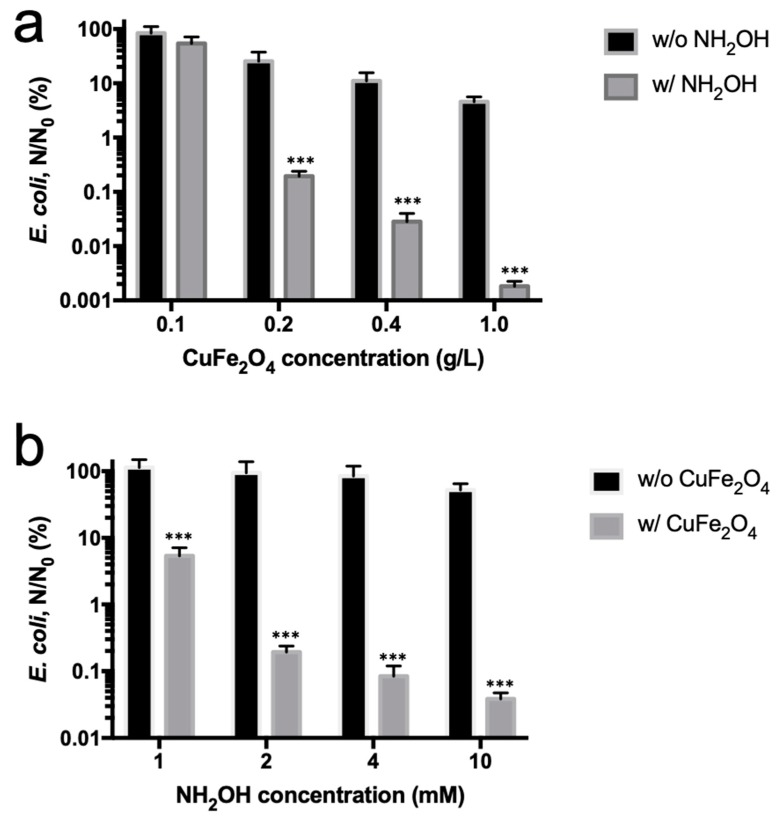
Effect of (**a**) CuFe_2_O_4_ and (**b**) NH_2_OH concentration on *E. coli* inactivation by CuFe_2_O_4_/NH_2_OH reaction. During reactions, (**a**) 0.1–1 g/L CuFe_2_O_4_, 2 mM NH_2_OH, and 10^8^ CFU/mL *E. coli* cells in 10 mM MOPS buffer at pH 7 were used; (**b**) 0.2 g/L CuFe_2_O_4_, 1–10 mM NH_2_OH, and 10^8^ CFU/mL *E. coli* cells in 10 mM MOPS buffer at pH 7 were used. *** *p* < 0.001.

The effect of CuFe_2_O_4_ nanoparticles or NH_2_OH concentrations was further investigated. At first, the CuFe_2_O_4_ nanoparticle concentration varied between 0.1 and 1 g/L, while NH_2_OH concentration was fixed at 2 mM. The results showed that, at a concentration of 0.1, 0.2, 0.4, and 1 g/L CuFe_2_O_4_ nanoparticles, the *E. coli* inactivation rate was 0.27-, 2.71-, 3.55-, and 4.74-log, respectively (Figure 5a), suggesting a dose-dependent CuFe_2_O_4_ nanoparticle-induced reduction of bacterial viability. This is presumably because more exposed Cu(I) species mediated by NH_2_OH reduction acted as a highly potent antibacterial agent [32,33]. We subsequently evaluated the effect of NH_2_OH concentration. A total of 1 to 10 mM NH_2_OH was used to mix with 0.2 g/L CuFe_2_O_4_ nanoparticle for bactericidal assays. It was shown that at 1, 2, 4, and 10 mM NH_2_OH, 1.27-, 2.71-, 3.07-, and 3.41-log *E. coli* inactivation rate was obtained (Figure 5b). The plateaued enhancement of cell inactivation by increased NH_2_OH doses was perhaps because of the fact that the majority of copper species on 0.2 g/L CuFe_2_O_4_ were reduced by 4 mM NH_2_OH, and the further increase in NH_2_OH concentration did not improve copper reduction. Such a trend has also been observed in other studies [7].

### 3.3. Reduction of Surface Cu(II) into Cu(I) by Hydroxylamine

It has been previously reported that NH_2_OH was able to efficiently transform Cu(II) ion into Cu(I) ion, which shows approximately 100–1000-fold enhancement in terms of antibacterial activity [7,33]. Because CuFe_2_O_4_ also owns Cu(II) species exposed onto the nanoparticle surface, we thus explored if the highly bactericidal Cu(I) species was formed by NH_2_OH reduction. XPS (X-ray photoelectron spectroscopy) was utilized to detect the electronic properties of elements on the surface of CuFe_2_O_4_ nanoparticles. The electronic properties of CuFe_2_O_4_ nanoparticles were tested before and after reduction by NH_2_OH.

**Figure 6:**
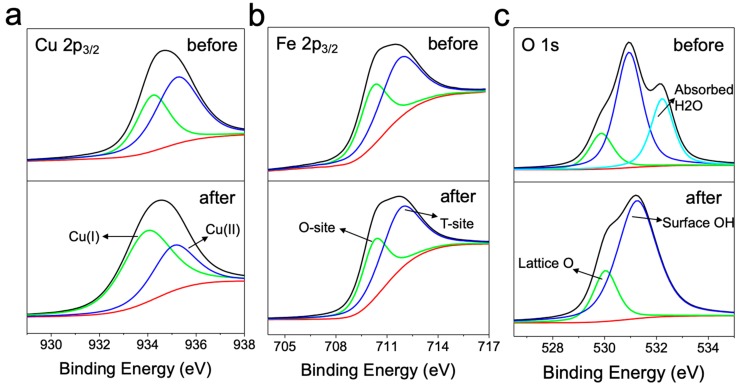
X-ray photoelectron spectroscopy (XPS) characterization of synthesized CuFe_2_O_4_ nanoparticles before (upper panel) and after (lower panel) reduction by NH_2_OH. (**a**) Cu 2p_3/2_, (**b**) Fe 2p_3/2_, and (**c**) O 1s peaks were deconvoluted.

XPS results are shown in Figure 6. It was observed that Fe 2p_3/2_ peaks almost did not show any detectable change in either octahedral or tetrahedral site Fe(III) species (Figure 6b), suggesting that iron species may not participate in the redox evolution of CuFe_2_O_4_/NH_2_OH reaction. Besides, it was shown in Figure 6c that, after addition of NH_2_OH, the surface adsorbed H_2_O molecules were diminished, primarily because NH_2_OH repulsed H_2_O molecules away to approach the CuFe_2_O_4_ surface. Interestingly, the addition of NH_2_OH drastically changed the speciation of copper (i.e., Cu(I) and Cu(II)) on CuFe_2_O_4_ nanoparticle surfaces based on Cu 2p_3/2_ deconvolution results (Figure 6a). Specifically, the fraction of Cu(I) before and after CuFe_2_O_4_/NH_2_OH reaction was 27.4% and 75.2%, respectively (Table 1). The remarkable increase of Cu(I) species was primarily because of the reductive action of NH_2_OH. Besides, CuFe_2_O_4_/NH_2_OH reaction also mediated a significant change in O 1s electronic property. For example, O 1s of CuFe_2_O_4_ was mainly composed by lattice O (28.6%), surface OH (66%), and absorbed H2O (47.4%). However, after reaction with NH_2_OH, the components of O 1s on CuFe_2_O_4_ nanoparticle surface became lattice O (32.5%) and surface OH (67.5%), whereas the surface adsorbed H_2_O molecules disappeared (Table 1). This is probably because NH_2_OH might need to repel surface adsorbed H_2_O molecule before accessing the reactive center on CuFe_2_O_4_ nanoparticle. Overall, the results indicated that Cu(I) fraction was successfully increased on the surface of CuFe_2_O_4_ after NH_2_OH reduction, and the transformed nascent Cu(I) species is supposed to play a major role in *E. coli* inactivation.

### 3.4. Bactericidal Action by CuFe_2_O_4_/NH_2_OH Reaction

**Figure 7:**
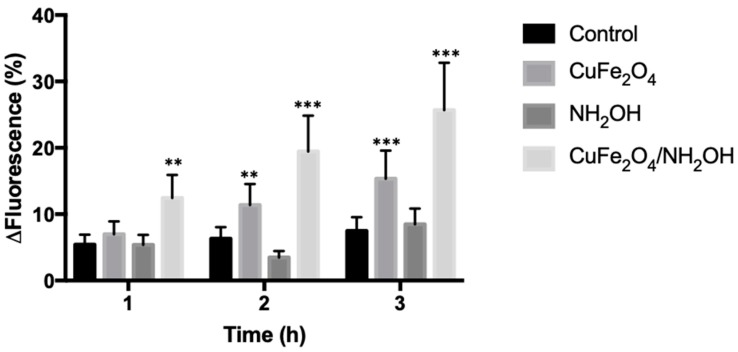
HPF (3’-p-(hydroxyphenyl) molecular probe fluorescence change of *E. coli* cells by CuFe_2_O_4_/NH_2_OH and related controls. Excitation/emission at 490/515 nm was used for fluorescence determination. Control: No addition of CuFe_2_O_4_ or NH_2_OH. During reactions, 10 µM HPF probe, 0.2 g/L CuFe_2_O_4_, 2 mM NH_2_OH, and 10^8^ CFU/mL *E. coli* cells in 10 mM MOPS buffer at pH 7 were used. ** *p* < 0.01, *** *p* < 0.001.

We were interested in understanding the molecular biology mechanism associated with the bactericidal action of CuFe_2_O_4_/NH_2_OH reaction. It has been reported that Cu(I) is a strong complexing and denaturing agent for functional proteins in particular membrane proteins [44,45,46,47,48]. In fact, the antibacterial potency of Cu(I), which is called contact-killing [47,48], is significantly higher than other well-established heavy metals such as silver. Although the exact bactericidal actions of Cu(I) are unclear, it is widely accepted that it increases the oxidative stress inside the cell [44,47,48]. We thus attempted to evaluate the ROS content—A direct oxidative stress indicator – with a fluorescent probe [49,50]. The results suggested that the incubation of CuFe_2_O_4_ and CuFe_2_O_4_/NH_2_OH with *E. coli* cells could increase the ROS content by comparison with the control (Figure 7). Specifically, after incubation for 3 h, the fluorescence change for the control, CuFe_2_O_4_, NH_2_OH, and CuFe_2_O_4_/NH_2_OH was 7.48%, 15.34%, 8.49%, and 25.69%, respectively. In addition, the CuFe_2_O_4_/NH_2_OH treatment mediated a more significant increase in ROS content than the CuFe_2_O_4_ treatment, indicating that the generated Cu(I) species is more powerful in producing oxidative stress, in agreement with previous literatures [44,47,48]. 

**Figure 8:**
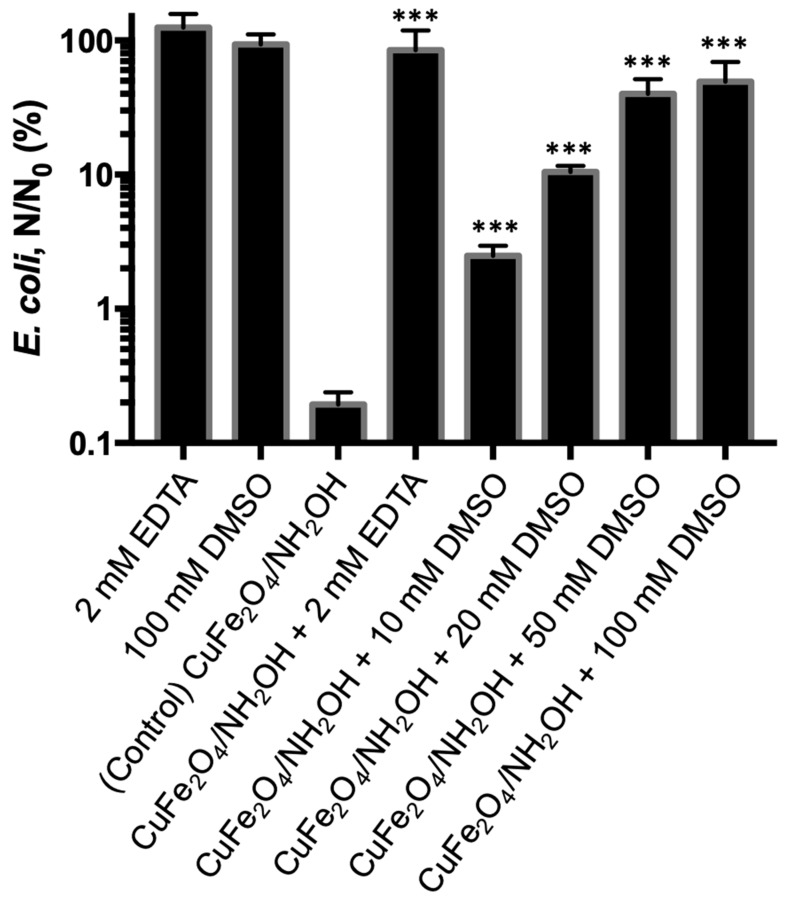
*E. coli* inactivation by CuFe_2_O_4_/NH_2_OH after addition of EDTA and DMSO scavengers and related controls. During reactions, 0.2 g/L CuFe_2_O_4_, 2 mM NH_2_OH, and 10^8^ CFU/mL *E. coli* cells in 10 mM MOPS buffer at pH 7 were used. EDTA of 2 mM and DMSO of 10–100 mM were added in the above bacterial solution. *** *p* < 0.001.

**Figure 9:**
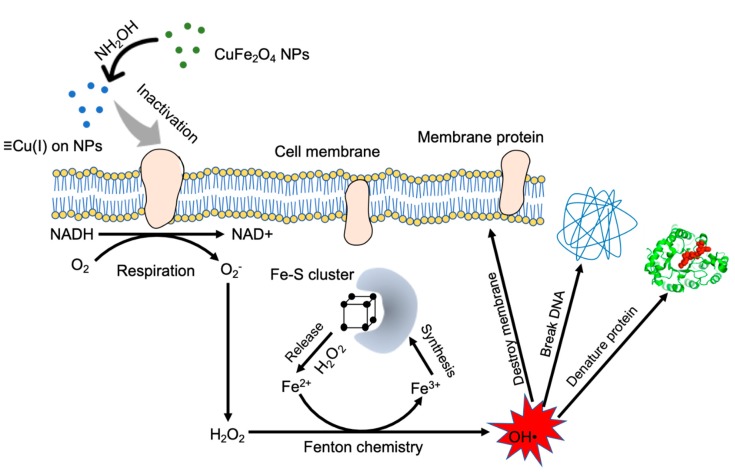
Proposed mechanism of CuFe_2_O_4_/NH_2_OH reaction inactivating *E. coli* cells. Generated Cu(I) species on nanoparticle surface after NH_2_OH reduced CuFe_2_O_4_ inactivates membrane proteins, leading to generation of H_2_O_2_. The disruption of the Fe–S cluster by H_2_O_2_ releases free Fe^2+^ ions, which catalyzes the Fenton chemistry to convert H_2_O_2_ into HO• (a reactive oxygen species). The HO• inactivates *E. coli* cell through destroying the membrane structure, breaking DNA, and denaturing functional proteins. NPs, nanoparticles.

The interaction between Cu(I) species and *E. coli* cell was further explored. At first, we added 2 mM EDTA as a complexing reagent to block the effective binding of copper species to membrane proteins, and found that the bacterial inactivation was negligible (0.07-log) (Figure 8). The above results verified the bactericidal role of copper species in CuFe_2_O_4_. We further investigated if the bacterial inactivation could be alleviated by adding a ROS scavenger. DMSO was used as a ROS scavenger [51,52,53], and a different DMSO concentration (10–100 mM) was used. It was found that the addition of DMSO could suppress the bactericidal potency of CuFe_2_O_4_/NH_2_OH reaction. In detail, after the addition of 10, 20, 40, and 100 mM DMSO, *E. coli* inactivation efficiency by CuFe_2_O_4_/NH_2_OH reaction was 1.60-, 0.98-, 0.40-, and 0.31-log, respectively. The results clearly indicated that ROS generation was the major reason accounting for bacterial inactivation in our system, which is in good accordance with other reports [7,15]. A detailed mechanistic illustration is shown in Figure 9.

### 3.5. Recycling Assay of Copper Ferrite Nanoparticle

**Figure 10:**
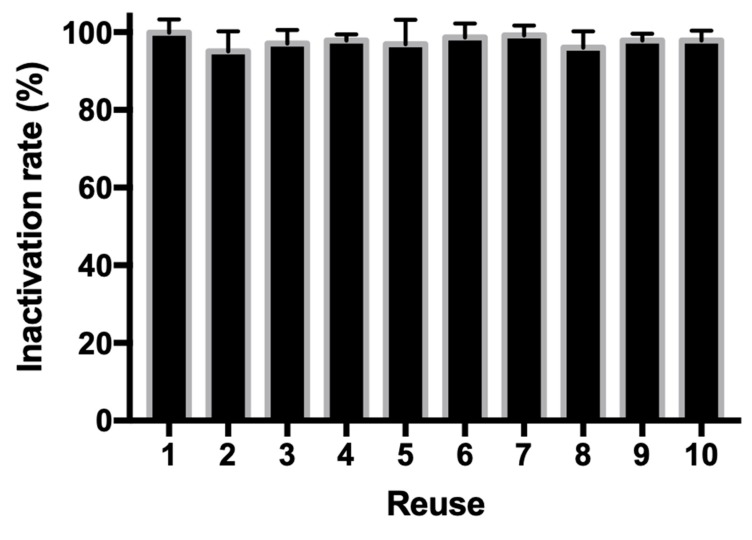
Inactivation of *E. coli* by reused CuFe_2_O_4_ nanoparticles and addition of NH_2_OH. During reactions, 0.2 g/L fresh or reused CuFe_2_O_4_, 2 mM NH_2_OH, and 10^4^ CFU/mL *E. coli* cells in 10 mM MOPS buffer at pH 7 were used.

An attractive advantage associated with heterogeneous water disinfection system is that the antibacterial agents could be reused for multiple rounds. To test if CuFe_2_O_4_ nanoparticle could be reused in our developed water disinfection platform, after each round of bactericidal assay, the nanoparticles were centrifuged and collected for a subsequent round of analysis. Then, 10^4^ CFU/mL exponential phase *E. coli* cells were used to simulate real water bodies. Figure 10 shows that, during the 10 rounds of tests, the reused CuFe_2_O_4_ nanoparticles showed a steady antibacterial efficiency, from 96.1% to 99.9%, indicating that the proposed CuFe_2_O_4_/NH_2_OH antibacterial platform could be used for water treatment industry with a low cost. Besides, it is worth to mention that CuFe_2_O_4_ nanoparticles (NPs) have a strong magnetic property and could be collected by magnetic attraction. This property further simplifies the reuse procedure in industry because magnetic enrichment and collection has been well established.

## 4. Conclusions

In this study, we showed that the antibacterial capability of CuFe_2_O_4_ nanomaterial could be significantly enhanced after addition of hydroxylamine. This was because surface Cu(II) species was successfully reduced to Cu(I), as evidenced by XPS. Cu(I) has a much stronger binding affinity and reduction capability to functional proteins on bacterial cell membrane than Cu(II) species, leading to a contact-killing phenomenon. It is worth noting that the bacterial death caused by CuFe_2_O_4_/NH_2_OH reaction was mainly because of the Cu(I) species on the nanoparticle surface, rather than that dissolved in solution. This implies that the minimum leaching of CuFe_2_O_4_ nanoparticle guarantees its safe application in the water disinfection industry. Besides, NH_2_OH has also been widely used in water treatment, and meets the criteria of public drinking water safety. 

Further, the bactericidal mechanism of CuFe_2_O_4_/NH_2_OH reaction towards *E. coli* was revealed with multiple molecular approaches. The results indicated that ROS content is elevated inside the cell, which might impair vital cellular components and cause leakage, presumably accounting for the death of *E. coli* cells. In addition, CuFe_2_O_4_ nanoparticles were reused for several rounds in our study, delivering uncompromised *E. coli* inactivation performance. In conclusion, considering the low cost of the chemicals and negligible secondary contamination concern, these results demonstrated that the generation of Cu(I) species immobilized on CuFe_2_O_4_ nanoparticles after reduction by NH_2_OH is a viable option for water pathogens’ disinfection.

## Figures and Tables

**Table 1:** Species distribution of Cu 2p, Fe 2p, and O 1s on CuFe_2_O_4_ nanoparticle surface before and after reduction by NH_2_OH.

	Cu 2p		Fe 2p		O 1s		
	Cu(II)	Cu(I)	O-Site Fe(III)	T-Site Fe(III)	Lattice O	Surface OH	Absorbed H_2_O
Before	72.6%	27.4%	45.5%	54.5%	28.6%	66%	47.4%
After	24.8%	75.2%	42.4%	57.6%	32.5%	67.5%	n/a
